# Think Fungus—Prevention and Control of Fungal Infections

**DOI:** 10.3201/eid1910.131092

**Published:** 2013-10

**Authors:** Mary E. Brandt, Benjamin J. Park

**Affiliations:** Centers for Disease Control and Prevention, Atlanta Georgia, USA

**Keywords:** emerging fungal infections, fungal infections, mycotic diseases, fungi, prevention, control

Reports of human infections with environmental fungi are on the increase throughout the world. Many of these reports describe infections caused by new agents, as well as by traditional agents with new virulence factors or new mechanisms of infection. Fungal infections historically have been underrecognized and difficult to detect, and treatment options are poor.

The reasons for their emergence are likely multifactorial. The advent of medical progress—including the wide use of medical hardware, such as central lines; successful management of immunosuppression in patients with transplanted organs; and immunomodulatory agents for treating underlying diseases from cancer to rheumatoid arthritis—has contributed to the increase in fungal infections in immunocompromised hosts. Risk factors such as changes in land use, seasonal migration, international travel, extreme weather, and natural disasters, and the use of azole antifungal agents in large-scale agriculture are believed to underlie many of the increases in community-acquired fungal infections.

Because fungal infections are frequently underrecognized and difficult to detect, one of the largest gaps in our understanding of their epidemiology is determining the incidence of disease. In an article in this issue of Emerging Infectious Diseases, Sondermeyer et al. document the incidence and cost of hospitalizations in California caused by Valley fever (coccidioidomycosis), a fungal infection endemic to the southwestern United States and parts of Latin America (*1*). This article reports that during 2000–2011, there were >25,000 Valley fever–associated hospitalizations in California and >$2 billion in hospital costs. The rate of hospitalizations increased over the study period from 2.3 to 5.0 cases/100,000 population, a finding that supports other recent publications documenting the increasing incidence of Valley fever in the United States (*2*,*3*). Although the reasons for this increase are not well understood, the practical effect of this increasing incidence has been seen in many settings, including the California state prison system. Recently, a federal court ordered the prison system to move prisoners believed to be at high risk for Valley fever (including Blacks, persons >55 years of age, and persons with preexisting diabetes) out of 2 prisons in the San Joaquin Valley, which is the region in California to which coccidioidomycosis is endemic.

Fungal diseases also appear to be emerging beyond their traditionally described borders for reasons that are not entirely understood. One article in this issue reports the incidence of *Cryptococcus gattii* disease, once believed to be restricted to tropical regions, but which is now found in locations as disparate as Vancouver Island, Canada and parts of the southeastern United States (*4*). Although this organism is genetically related to *C. neoformans*, a cause of meningitis in HIV-infected persons, *C. gattii* is frequently associated with a different spectrum of disease, prominently pneumonia. In another article in this issue, Nucci et al. report an increase in incidence of community-associated *Fusarium* spp. infections in a cancer ward in Brazil (*5*). In this study, *Fusarium* spp. caused an increase in invasive infections, which usually started as skin or nail infections, in immunocompetent and immunosuppressed patients. Although the root cause was not determined, speculation has centered on changes in land use patterns and agricultural practices in Brazil. Also in this issue, a novel agent of fungemia, *Candida auris*, is reported as having been detected in India (*6*). All isolates reported in this article were resistant to the antifungal agent fluconazole, which is concerning because fluconazole is frequently the first-line treatment for invasive *Candida* spp. infections in many countries. Finally, an article by Fong et al. provides serologic evidence that *Pneumocystis jirovecii* may be transmitted between patients and providers in the health care setting, a finding that could affect future infection control policies (*7*).

Because most invasive fungal infections have high mortality rates, reducing the incidence of these diseases often relies on rapid and specific diagnostics, effective antifungal drugs, novel immunotherapeutic strategies, and adherence to infection control and sterility practices. Recently, we have seen examples of successes and failures in this area. In regions with high HIV prevalence, use of novel lateral-flow diagnostic tests for cryptococcal disease has opened the door to systematic screening and point-of-care testing in asymptomatic persons with low CD4 cell counts and may result in reduction of deaths caused by this disease (*8**,**9*). Conversely, recent contamination of a widely distributed injectable steroid medication with fungal organisms, particularly the black mold *Exserohilum rostratum*, caused the largest health care–associated outbreak in the United States; as of July 1, 2013, there have been 749 cases of meningitis and related infections among persons in 20 states and 61 deaths (*10**,**11*). Swift public health actions, including notification of patients and providers, led to rapid clinical assessments and institution of antifungal therapy among infected persons, thereby reducing the mortality rate and effects of this disease (*12*).

Broader control of fungal exposures in the community can also be improved by awareness, especially education regarding high-risk practices and activities. Outbreaks of histoplasmosis linked to construction and cleaning activities in places contaminated with bird or bat guano have led to production of educational materials describing how risk can be mitigated (*13*). Furthermore, recent advances in whole-genome sequencing are being explored to suggest novel vaccine and diagnostic targets for the agent of Valley fever (*14*).

Fungal infections remain serious and underappreciated causes of illness and death. Much can be done to prevent the consequences of these infections, although environmental exposure to these agents may not be entirely avoidable in the community. Continued public health efforts toward defining, characterizing, and tracking the emergence of fungal infections can help to focus studies on priority infections and settings. Future translational research is urgently needed to develop novel diagnostics, vaccines, and treatments as more is learned about the pathogenesis of fungal infections and the biology of fungal agents.
